# Prognostic value of neuron-specific enolase after cardiac arrest and targeted temperature management

**DOI:** 10.1186/cc14517

**Published:** 2015-03-16

**Authors:** K Streitberger, C Leithner, CJ Ploner, C Storm

**Affiliations:** 1 Charité Universitätsmedizin, Berlin, Germany

## Introduction

The serum concentration of neuron-specific enolase (NSE) has been established as a highly specific predictor of poor outcome after cardiac arrest. Recent studies have indicated that patients treated with targeted temperature management at 33°C for 24 hours may have good outcome despite NSE serum concentrations considerably higher than the cutoff established for normothermic patients. The threshold above which survival with good outcome becomes very unlikely, its positive predictive value and sensitivity for prediction of poor outcome have not been established in this patient group. Furthermore, a threshold below which hypoxic encephalopathy may be largely excluded has not been determined.

## Methods

From 2006 through 2014 we prospectively included in-hospital and out-of-hospital cardiac arrest patients treated with targeted temperature management at 33°C for 24 hours. The NSE serum concentration was determined 3 days after cardiac arrest and the outcome was assessed according to the Cerebral Performance Category (CPC) upon ICU discharge. CPC 1 to 3 was defined as good outcome and CPC 4 to 5 as poor outcome. Individual case review was performed in patients with good outcome despite very high NSE serum concentration and in patients with poor outcome despite very low NSE serum concentration.

## Results

Of 601 included patients, 309 (51%) had good outcome. An NSE serum concentration threshold of 90 μg/l predicted poor outcome with a positive predictive value of 0.98 and a sensitivity of 0.51. Three patients survived with good outcome despite an NSE serum concentration >90 μg/l. In two of these patients NSE elevations had been documented prior to cardiac arrest. One patient had a neuroendocrine tumor of the pancreas, the other patient suffered from encephalitis of unknown etiology and an osteomyelofibrosis. Potential confounders in the third patient were an ovarian carcinoma, the use of an intra-aortic balloon pump and blood transfusions shortly after cardiac arrest. Only 16 of 205 patients with NSE <17 μg/l had poor outcome, the majority of these patients died from causes other than hypoxic encephalopathy.

## Conclusion

In patients with cardiac arrest and targeted temperature management at 33°C, an NSE serum concentration of >90 μg/l strongly indicates poor outcome. NSE producing tumors, acute brain diseases, severe hematologic diseases, use of an intra-aortic balloon pump and blood transfusions need to be considered as potential confounders. An NSE serum concentration of <17 μg/l largely excludes hypoxic encephalopathy incompatible with re-awakening.

